# Magneto-Chemotaxis in Sediment: First Insights

**DOI:** 10.1371/journal.pone.0102810

**Published:** 2014-07-17

**Authors:** Xuegang Mao, Ramon Egli, Nikolai Petersen, Marianne Hanzlik, Xiuming Liu

**Affiliations:** 1 College of Geographical Sciences, Fujian Normal University, Fuzhou, China; 2 Central institute for Meteorology and Geodynamics, Vienna, Austria; 3 Department of Earth and Environmental Sciences, Ludwig-Maximilians University, Munich, Germany; 4 Chemistry Department, Munich Technical University, Munich, Germany; 5 Department of Environment and Geography, Macquarie University, Sydney, Australia; University of California, Irvine, United States of America

## Abstract

Magnetotactic bacteria (MTB) use passive alignment with the Earth magnetic field as a mean to increase their navigation efficiency in horizontally stratified environments through what is known as magneto-aerotaxis (M-A). Current M-A models have been derived from MTB observations in aqueous environments, where a >80% alignment with inclined magnetic field lines produces a one-dimensional search for optimal living conditions. However, the mean magnetic alignment of MTB in their most widespread living environment, i.e. sediment, has been recently found to be <1%, greatly reducing or even eliminating the magnetotactic advantage deduced for the case of MTB in water. In order to understand the role of magnetotaxis for MTB populations living in sediment, we performed first M-A observations with lake sediment microcosms. Microcosm experiments were based on different combinations of (1) MTB position with respect to their preferred living depth (i.e. above, at, and below), and (2) magnetic field configurations (i.e. correctly and incorrectly polarized vertical fields, horizontal fields, and zero fields). Results suggest that polar magnetotaxis is more complex than implied by previous experiments, and revealed unexpected differences between two types of MTB living in the same sediment. Our main findings are: (1) all investigated MTB benefit of a clear magnetotactic advantage when they need to migrate over macroscopic distances for reaching their optimal living depth, (2) magnetotaxis is not used by all MTB under stationary, undisturbed conditions, (3) some MTB can rely only on chemotaxis for macroscopic vertical displacements in sediment while other cannot, and (4) some MTB use a fixed polar M-A mechanisms, while other can switch their M-A polarity, performing what can be considered as a mixed polar-axial M-A. These observations demonstrate that sedimentary M-A is controlled by complex mechanical, chemical, and temporal factors that are poorly reproduced in aqueous environments.

## Introduction

Magnetotactic bacteria (MTB) are a polyphyletic group of bacteria living in chemically stratified freshwater and marine environments within the so-called oxic-anoxic interface (OAI) [1], [2]. MTB contain magnetite (Fe_3_O_4_) or greigite (Fe_3_S_4_) single-domain crystals, usually arranged in chains, whose magnetic moment ensures a passive alignment of the whole cell with the Earth magnetic field. Alignments >80% make MTB cells swim straight along magnetic field lines when observed under the optical microscope: this phenomenon is called magnetotaxis [1], [3]. The swimming direction (parallel or antiparallel to the magnetic field vector ***B***) is determined by the sense of flagellar rotation, which is controlled mainly by chemical factors, such as oxygen concentration and redox potential [5]–[7], and, to a certain extent, by physical stimuli such as light [8], [9] and physical contact [10]. The MTB response to oxygen gradients in a magnetic field has been called magneto-aerotaxis (M-A); here we use this term in a broad sense to indicate the combination of passive magnetic alignment and chemical control of flagellar rotation.

The confinement of magneto-aerotaxis to displacements along inclined magnetic field lines that intersect horizontally stratified environments reduces a 3D search for optimal living conditions to a more efficient 1D search [1], [11], providing MTB with a so-called magnetotactic advantage [12]. Two different magneto-aerotaxis mechanisms, called axial and polar magnetotaxis, have been observed in MTB cultures. Axial magnetotaxis is based on a temporal sensory mechanism that determines the swimming direction according to the sensed chemical gradient (e.g. *Magnetospirillum magnetotacticum*). Polar magnetotaxis, on the other hand, appears to be controlled by a threshold mechanism, so that cells swim consistently along a direction determined by the oxygen concentration, regardless of existing gradients (e.g. *Magnetococcus marinus* MC-1) [5]. In hanging drop assays with MTB grown in the Northern hemisphere, polar magnetotaxis is characterized by most cells swimming consistently parallel to the magnetic field, i.e. towards the magnetic North (N-seeking, or NS cells) and only few cells (<0.1%) swimming along the opposite direction (S-seeking, or SS cells). Because ***B*** points downwards in the Northern hemisphere, this behaviour is consistent with a downward displacement that enables cells to escape high oxygen concentrations in normally stratified environments. Axial magnetotaxis, on the other hand, appears as a random sequence of swimming direction reversals in hanging drop assays, due to the lack of a defined chemical gradient.

In most freshwater and marine environments, the OAI is located within the upper 3–10 cm of sediment, where living MTB populations have been systematically found (e.g [13], [14]). M-A in sedimentary environments might be affected significantly by (1) mechanical interactions with sediment particles acting as additional randomizing forces against magnetic alignment, and (2) solid interfaces adding local perturbations to macroscopic chemical stratifications. Unfortunately, while realistic living conditions for natural MTB populations can be reproduced in the laboratory with sediment microcosms [15], [16], M-A has never been studied outside liquid MTB cultures, due to the impossibility of performing direct MTB observations in sediment. Recently, the mean alignment of *Candidatus Magnetobacterium bavaricum* with Earth-like magnetic fields has been indirectly measured in sediment microcosms [17], where it does not exceed 1%. Accordingly, MTB displacement in sediment is described by a slightly biased random walk, rather than straight paths, with magnetotaxis overcoming the random diffusive component of the swimming path only over macroscopic distances estimated to exceed 1 mm [17]. Microscopic displacements, required for instance to overcome natural sediment mixing and avoid passive diffusion away from the preferred living depth, are therefore not expected to be assisted by the Earth magnetic field. Within this context, the role of magnetotaxis in sediment becomes less obvious than suggested by the 1D displacement model in water. Furthermore, simple M-A tests with wild-type MTB, such as hanging drop assays performed under strictly anoxic conditions and long-term observations of natural MTB populations in magnetically shielded microcosms, do not appear to support the current understanding of M-A.

In order to clarify the role of M-A in sediment, we have undertaken a systematic series of experiments aimed at investigating vertical migration and resilience of natural MTB populations in sediment subjected to selected favourable, unfavourable, or indifferent magnetic field configurations. Our results clarify the circumstances under which M-A supports navigation in sediment and reveal important differences between two MTB populations characterized by polar magnetotaxis.

## Materials and Methods

### 1. Microcosms

**Figure 1:**
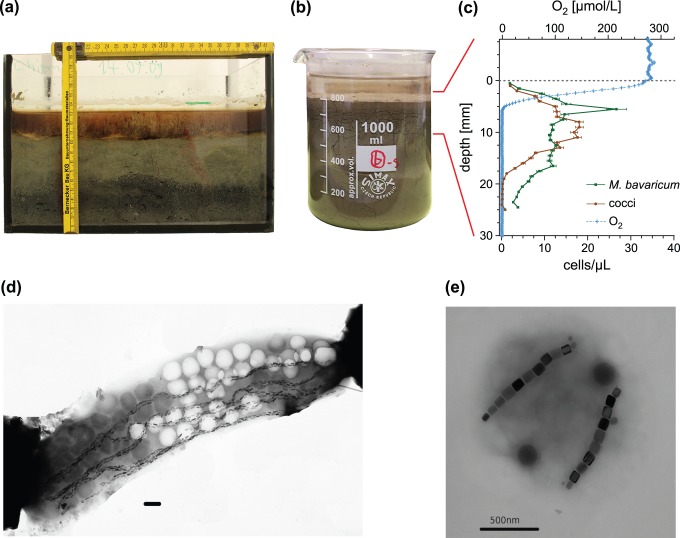
Microcosms and MTB characterization. (A) 30×20×20 cm glass aquarium filled with sediment. (B) 1 L sediment microcosm. (C) Oxygen profile measured after 7 days stabilization, and mean MTB profiles obtained from the average of 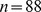 minicores. Error bars correspond to 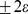, where 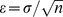 is the standard error estimated from the standard deviation *σ* of *n* individual minicore profiles. (D) Transmission electron micrograph (TEM) image of *M. bavaricum* containing five bundles of tooth-shaped magnetosomes and several empty (light) and filled (dark) sulfur inclusions. The horizontal bar corresponds to 0.5 µm. (E) TEM image of a coccus containing two chains of prismatic magnetosomes. The horizontal bar corresponds to 0.5 µm.

All experiments have been performed on microcosms prepared with surface sediment from Lake Chiemsee, southern Germany [14], [18]. Sediment has been collected with permission of the local authorities (Prien am Chiemsee municipality) using a bottom grab sampler on a boat of the local fire brigade, and transported to the laboratory, where it was transferred into several 30×20×20 cm glass aquaria (Figure 1a). Aquaria were allowed to stabilize for few weeks under constant laboratory conditions. Sediment was covered by 3–5 cm water and evaporation was steadily compensated by adding small amounts of distilled water. Microcosms have been prepared by transferring sediment slurry from aquaria into 1 L glass beakers (Figure 1b). The slurry was allowed to rest for at least 7 days in order to rebuild a stationary chemical stratification, characterized by an OAI located ∼5 mm below the sediment-water interface (Figure 1c). Chemical stratification was controlled prior to every microcosm experiment by measuring profiles of dissolved oxygen with a motorized microprobe (OX50 from Unisense).

### 2. MTB characterization

Two relatively stable MTB populations occur in Chiemsee sediment after laboratory storage: *M. bavaricum* (Figure 1d), a large (∼10 µm long), rod-shaped bacterium swimming at ∼40 µm/s [16], [19], [20], and unspecified cocci (∼1 µm diameter, Figure 1e) with up to 1 mm/s swimming velocities [21]. The identification of *M. bavaricum* (MBV) and magnetotactic cocci (MCC) under the optical microscope is based exclusively on their size and swimming behaviour (video S1). The two groups of bacteria appear to be homogeneous with respect to all experimental results presented here; although MCC might be phylogenetically heterogeneous [22]. Both groups perform polar magnetotaxis, as seen from consistently NS swimming directions in hanging drop assays. Observed NS:SS proportions were systematically >1000∶1, and the existence of few incorrectly polarized cells is believed to be essential for allowing MTB to survive geomagnetic field reversals [23]. Magnetotactic spirilla and vibrios could be observed occasionally, but have not been considered for this study due to their erratic occurrence.

Vertical distributions of the two MTB populations in microcosm sediment represent the fundamental characterization tool for most experiments presented in this paper, and have been obtained as follows. Mini-cores of the uppermost ∼3 cm of unconsolidated sediment have been sampled with a drinking straw (diameter 5 mm), and sliced in 1 mm increments. Each slice was diluted with distilled water (200 µL) and prepared for a hanging drop assay with a special optical microscope equipped with Helmholtz coils for producing controlled magnetic fields in the objective plane ([17]; text S1). In order to overcome natural MTB population heterogeneities, results from at least 7 minicores have been averaged to obtain a single profile. Mean MBV and MCC vertical distributions in undisturbed microcosms exposed to the Earth magnetic field are shown in Figure 1c. The MCC distribution is unimodal with ∼90% cell counts comprised between 3 and 15 mm, while MBV extends to greater depths (>25 mm), according to a bimodal distribution. Similar MBV distributions have been reported for other microcosms prepared with the same type of sediment [16].

### 3. Hanging drop assays under anoxic conditions

In order to test the capability of MBV and MCC to switch swimming direction under strictly anoxic conditions, as expected from the M-A model, a sediment microcosm was placed inside a glove box, together with the microscope apparatus used for the hanging drop assay (text S1). The glove box was steadily flushed with nitrogen gas bubbled through a FeCl_2_ solution for residual oxygen removal. After 4 weeks, oxygen dropped below detection threshold in sediment as well as the water column above it. Hanging drop assays have been performed directly inside the glove box with sediment samples taken at 2–25 mm below the sediment-water interface, using oxygen-free water from the microcosm. In order to avoid possible phototactic interferences, MTB were allowed to accumulate at the edges of the hanging drop in complete darkness for 20 minutes.

### 4. Resilience experiments

Long-term resilience experiments have been performed with a sediment-filled aquarium placed at the centre of three orthogonal ∼1×1 m Helmholtz coil pairs connected to precision power supplies for generating controlled, homogeneous fields over the aquarium volume. The experiment, which lasted for >2 years, has been performed with the following field settings. During the first 92 days, the aquarium was exposed to the laboratory field (∼44 µT with 71° downward inclination), with no current flowing through the Helmholtz coils. The laboratory field was then carefully compensated with the Helmholtz coils, in order to obtain nearly zero-field conditions for the next 194 days. Natural fluctuations of the residual field were monitored at regular time intervals and never exceeded ±1.5 µT. A preferred magnetic direction was not available to MTB during this time, because residual field fluctuations are effectively unbiased (text S1). The natural field was re-established at the end of this experiment for 138 days. Finally, during the last 98 days, the aquarium was exposed to a ∼120 µT vertical field generated by the Helmholtz coils. The field direction was reversed every 24 hours with an electronic commutator, so that MTB were exposed to upwards and downwards pointing fields every second day. This experimental setup aimed at testing the resilience of MTB population against field reversals, without exposing MTB to a consistent field direction favouring a particular magnetotaxis polarity. MTB profiles have been measured at regular time intervals during the whole experiment duration.

### 5. Observing vertical migration in sediment

**Figure 2:**
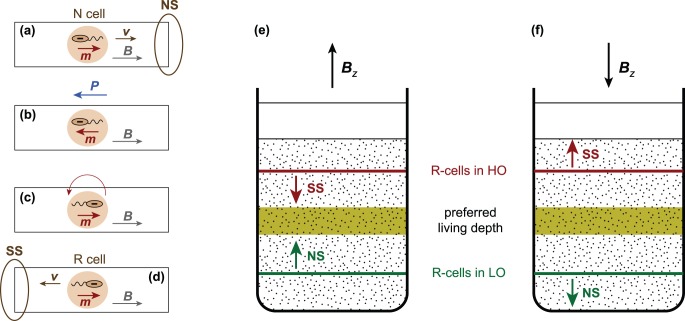
Production of ‘reversed’ MTB cells and vertical displacement detection in sediment. (A) ‘Normal’ MTB cell with polar magnetotaxis in the hanging drop assay. ***B*** is the magnetic field, ***m*** the cell magnetic moment, ***v*** the cell velocity. In oxygen-rich water, the cell is NS. (B) A short and strong magnetic pulse ***P*** antiparallel to ***B*** switches the cell magnetic moment before cell rotation takes place. (C) The switched cell rotates 180° so that ***m*** is again parallel to ***B***. (D) Because flagellar rotation has not changed, the cell with reversed magnetic moment (R-cell) becomes SS. (E) Microcosm containing two thin R-cell layers above and below the preferred living depth. If ***B*** is pointing upward, R-cells performing polar M-A are expected to move toward the preferred living depth (‘correct magnetotaxis’). (F) Same as (E) after reversing the direction of ***B***. R-cells are expected to move away from the preferred living depth (‘incorrect magnetotaxis’).

Vertical MTB profiles represent the dynamic equilibrium eventually reached by motile cells in a stationary environment: as such, they do not provide information about cell migration. Because direct MTB observations are not possible in sediment, an indirect method has been devised for tracking macroscopic vertical displacements, which is based on the overwhelming NS polarity of cells observed in hanging drop assays. This polarity is switched by applying a short (∼50 µs) magnetic pulse whose intensity (∼100 mT) is sufficient for reversing the magnetic moment of magnetosome chains before reorientation of the whole cell can take place [17]. After imparting a magnetic pulse against the steady field used for cell alignment, MTB rotate by 180° because of magnetic moment reconfiguration, thereby reversing their swimming direction. Accordingly, former NS cells become SS after a magnetic pulse (Figure 2a–d). Because flagellar rotation is unaffected by magnetic pulses, pulsed cells can be considered identical to the normal ones, except for the reversed magnetotactic polarity. In order to avoid confusion with the ‘natural’ SS behaviour controlled by flagellar rotation, we call ‘normal cells’ (N-cells) all polar MTB that have not been subjected to a magnetic pulse. These MTB behave as NS cells in the hanging drop assay, up to a negligible proportion of ‘wrongly polarized’ SS cells. On the other hand, polar MTB whose magnetic moments have been switched by a magnetic pulse behave as SS cells in the hanging drop assay and we call them ‘reversed cells’ (R-cells). Nearly equal amounts of NS and SS cells can be observed with hanging drop assays of pulsed sediment samples, instead of almost exclusively NS cells associated with untreated sediment. This result is expected if MTB living in sediment are almost randomly oriented, as reported in [17], because only cells whose magnetic moment from an angle of >90° to the pulsed field can be reversed.

N- and R-cells are always distinguishable in the hanging drop assay according to their NS and SS behaviours, respectively, regardless of the actual swimming polarity in sediment, which is controlled by chemical conditions. Therefore, if R-cells are created within a thin sediment layer by application of a magnetic pulse, the vertical migration of such cells can be monitored on sediment profiles by counting SS cells with the hanging drop assay. Because of the extremely low proportion of N-cells with SS behaviour, practically all SS cells retrieved at a certain depth in sediment must be R-cells coming from the thin layer where they have been created. Finding SS cells above (below) this layer means that R-cells have migrated upward (downward) in sediment. The migration direction is expected to depend on the position of R-cells relative to the preferred living depth, and on the vertical component of the local magnetic field (i.e. downward, as in the Northern hemisphere, or upward, as in the Southern hemisphere). The magnetic polarity of R-cells defines the following magnetotactic configurations: (1) ‘correct magnetotaxis’, if the field points upwards, (2) ‘incorrect magnetotaxis’, if the field points downwards, (3) ‘indifferent magnetotaxis’, if the field is horizontal, and (4) ‘no magnetotaxis’ in zero fields.

The role of sediment chemistry in determining the direction of vertical displacements is tested with two limit situations of the polar M-A model, i.e. (1) cells are located above their preferred living depth and therefore in a ‘high-oxygen’ state (HO), and (2) cells are located below their preferred living depth and therefore in a ‘low-oxygen’ state (LO). HO cells have the same swimming polarity observed with the hanging drop assay (i.e. NS for N cells and SS for R cells), while the opposite is expected for cells in a LO state. HO and LO states might depend on the whole sediment chemistry (e.g. O_2_, Eh, S^2−^), rather than oxygen alone; however, the situation of undisturbed sediment is analogous to the stratification of MTB cultures where magneto-aerotaxis has been observed [5]. Therefore, M-A observations of cultured MTB can be replicated in sediment microcosms, using R-cells as ‘tracer’ for macroscopic (>1 mm) vertical displacements. The influence of magnetotaxis on such displacements can be tested by comparing results obtained with identical microcosms subjected to different field configurations.

Natural situations for R-cells are reproduced with ‘correct magnetotaxis’ configurations where the local magnetic field points upwards (Figure 2e): HO R-cells are SS and move down in sediment, eventually switching to a LO state after crossing their preferred living depth, in which case they become NS and move upwards. This mechanism keeps MTB within their preferred living depths. The opposite situation, similar to a geomagnetic field reversal (‘incorrect magnetotaxis’), is created with a downward-pointing field (Figure 2f). In this case, R cells controlled by magnetotaxis move away from their preferred depth, and a population decline is expected over time due to sub-optimal living conditions. Finally, MTB must rely only on chemotaxis for vertical displacements in local magnetic field with zero vertical com­ponent (‘indifferent magnetotaxis’ or ‘no magnetotaxis’). The combination of R-cells in HO and LO states with different magnetic field configurations can therefore be used to assess the role of magnetotaxis in sediment without the need of direct observations.

A necessary condition for the correct interpretation of vertical migration observations performed with the abovementioned method is that R-cells should not form spontaneously over the experimental time when field polarity is favourable to them. Because the appearance of R-cells in sediment originally containing N-cells has been documented [1], [4], each experiment included an identical control microcosm without pulse-generated R-cells. All control microcosms displayed the usual NS:SS ratios >1000 over the whole experiment time (not shown), therefore excluding the spontaneous generation of R-cells.

### 6. Vertical migration experiments

Seven vertical migration experiments have been prepared according to the principles explained in section 5. In all experiments, microcosms consisted of two layers of identical sediment material, with only one layer (the top or the bottom one) containing R-cells. Microcosms have been prepared with the following procedure:

#### Microcosms with R-cells in bottom layer (M1–2 and M5–6 in Figure 3)

**Figure 3:**
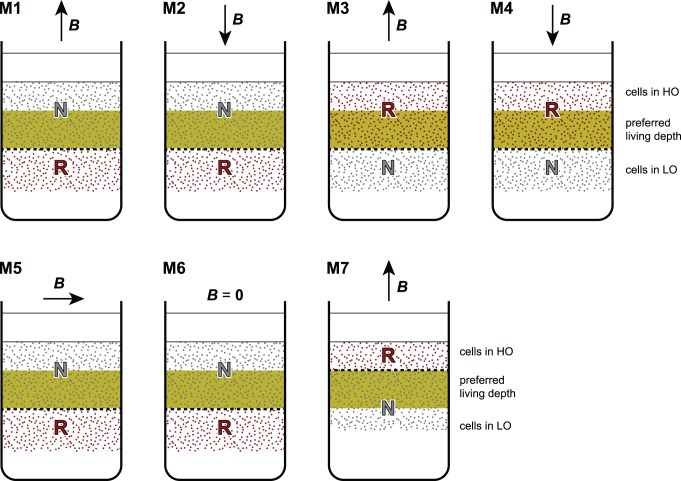
Sediment microcosms (M1–M7) used for vertical migration experiments. The microcosms consist of two sediment layers separated by a sharp boundary (dashed line) located above or below the preferred living depth (shaded area). Initially, only one layer contains R-cells, while N-cells occur in both layers. Vertical migration in a controlled field ***B*** is tracked by the appearance of R-cells in the other layer.

Stabilized microcosms have been repeatedly exposed to pulsed fields generated by a small pair of coils placed in proximity of the sediment surface. The coil pair was moved all over the sediment surface to create a homogeneous layer containing ∼50% R-cells. R-cells are concentrated within the uppermost 25 mm, where the pulse field is sufficiently strong for switching favourably oriented magnetic moments. Sediment containing exclusively N-cells has been subsequently deposited on the top of the existing microcosm sediment. In order to keep the interface between the two layers as sharp as possible, the second layer was formed by letting sediment material drop slowly from a sieve and deposit through the water column, in analogy with natural sedimentation (text S1).

#### Microcosms with R-cells in top layer (M3–4 and M7 in Figure 3)

These microcosms have been produced with the procedure described above, except for the fact magnetic pulses have been applied to the sediment material used for depositing the top layer, instead of the bottom layer. Consequently, R-cells are initially contained only in the top layer, where their concentration is ∼50%.

Immediately after their preparation, microcosms have been placed in a Helmholtz coil system pro­ducing a homogeneous ∼50 µT field directed vertically (experiments with ‘correct’ and ‘incorrect’ magnetotaxis), or horizontally (experiments with ‘indifferent’ magnetotaxis). Zero-field experiments have been performed by regulating the Helmholtz coils so, that the local Earth magnetic field was compensated up to a ∼0.5 µT residual field that changed in an unsystematic manner with time. The chemical stratification of freshly prepared microcosms is obviously disturbed, because both the top layer and part of the bottom layer are initially saturated with oxygen. As excess oxygen is consumed in sediment, the usual chemical stratification, with the OAI occurring ∼5 mm below the sediment-water interface, is re-established in 3–4 days. Therefore, all microcosms have been allowed to reach a new chemical equilibrium during ∼7 days. Oxygen profiles have been measured to ensure that conditions similar to the case of undisturbed microcosms were established before taking MTB profiles. Finally, MTB profiles have been measured with the procedure described in section 2, obtaining NS cell counts (N-cells) as well as SS cell counts (R-cells). Detection of SS cells outside the pulsed layer must be attributed to vertical migration, if the same phenomenon is not observed in control microcosms containing only N cells.

## Results

### 1. Hanging drop assays under anoxic conditions

**Figure 4:**
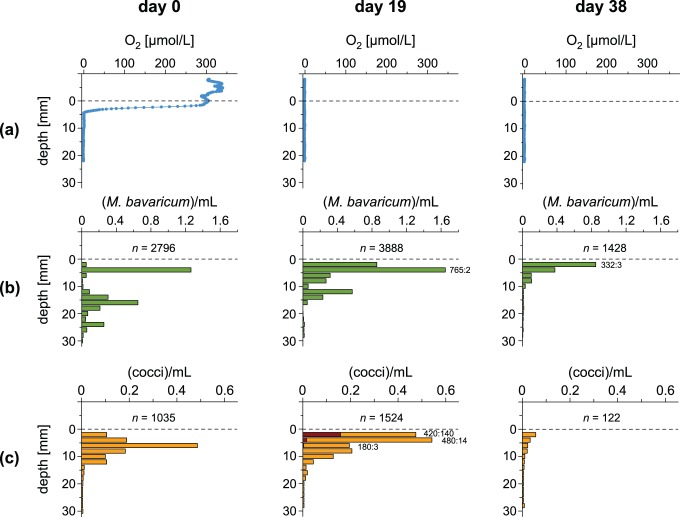
Evolution of oxygen concentration and cell counts in a microcosm under anoxic atmos­phere. (A) Oxygen profiles in water and sediment (0 = sediment/water interface), measured 0, 19, and 38 days after exposure to an oxygen-free atmosphere. (B–C) MBV and MCC cell concentrations as a function of sediment depth. Total MTB populations *n* (in 10^6^ cells/m^2^) and NS:SS proportions are given in each plot. Significant concentrations of SS cocci at day 19 are plotted as red bars.

Immediately after an oxygen-free atmosphere was established inside the glove box, the microcosm OAI moved upwards, reaching the sediment-water interface in 19 days and disappearing completely from the water column in 38 days (Figure 4a). MTB profiles moved up as well, following the OAI trend (Figure 4b–c), with maximum cell concentrations occurring at the sediment-water interface. On the other hand, MTB have never been found in the water column, while total cell counts decreased over time inside the sediment. Few SS cells have been observed in hanging drop assays prepared with sediment taken from the topmost 4 mm immediately after the OAI reached the water column, with up to 25% SS MCC-cells in a single profile. SS cell counts were extremely variable and decreased to <1% after 30 days.

A net predominance of SS cells in anoxic hanging drop assays, as expected for polar magnetotactic MTB in their LO state, could not be observed with this experimental setup. The transient increase of SS cell counts while the OAI was crossing the sediment-water interface, along with the fact that MTB did not follow the OAI into the water column, suggests that flagellar rotation might be controlled by additional factors besides oxygen concentration. Furthermore, strong mechanical stimulations and sudden chemical changes during preparation of the hanging drop assay can be considered analogous to sediment resuspension produced by a bioturbation event, and might therefore trigger a NS response useful to regain the preferred depth in sediment. Overall, results of hanging drop assays are inconclusive with respect to the characterization of sedimentary M-A.

### 2. Resilience experiments

**Figure 5:**
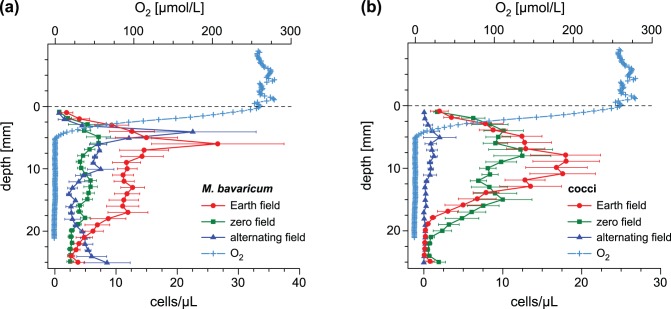
Mean MTB concentration profiles obtained during long-term exposures to different magnetic field configurations. Profiles refer to (A) *M. bavaricum*, and (B) cocci, during resilience experiments in the Earth magnetic field, zero field, and a daily switched vertical field. Error bars correspond to the standard deviation of individual minicors.

Resilience experiments have been used to monitor the reaction of undisturbed MTB populations to different magnetic field conditions corresponding to: (1) normal, downward-pointing laboratory field, (2) zero field, and (3) vertical field whose polarity is switched daily. The long experiment duration enabled collection of a large number of sediment profiles, which define the mean MBV and MCC concentration profiles shown in Figure 5. Integration of these profiles over sediment depth gives the mean number of cells per unit area, which can be considered as a measure for the total MTB population for the three abovementioned experimental conditions (Table 1).

**Table 1:** Total MTB populations in the Earth magnetic field, zero field and vertically alternating field.

Field setting	Earth field (*n* = 88)	Zero field (*n* = 94)	Alternating field (*n* = 47)
*M. bavaricum*	398±17	166±4 (rejected)	188±5 (rejected)
cocci	279±9	262±11 (not rejected)	13±1 (rejected)

Total MTB populations (in 10^6^ cells/m^2^± standard error) are obtained by integrating cell counts of *n* individual profiles sampled during exposure to the Earth magnetic field, zero field, and a vertical field whose direction was switched every day. Results of a Kolmogorov-Smirnov test with 95% confidence level are reported in parenthesis. The null hypothesis that zero or alternating fields did not affect MTB populations has been tested against the hypothesis of a population increase or decrease. In all cases, except for cocci in zero field, the population decreased significantly in zero or alternating fields.

Zero-field conditions produced a ∼50% decrease of the total MBV population by unchanged depth distribution. On the other hand, the total MCC population remained stable, but extended over a slightly larger depth range. Alternating field conditions produced the almost complete extinction of MCC, especially at >12 mm depths, and a ∼50% decrease of the total MBV population, whose depth distribution became markedly bimodal with two clearly distinct peaks at 4 mm and >25 mm. Population decrease in zero and alternating fields cannot be attributed to a natural declining trend, because complete recover was observed when the Earth magnetic field was re-established between the two time intervals. These results are difficult to reconcile with the M-A model, especially for the case of unchanged MCC concentrations in zero field (Table 1). At least in this case, it seems that magnetotaxis does not provide an advantage to MTB population living in undisturbed sediment.

### 3. Microcosm M3: R-cells at preferred depth, correct magnetotaxis

**Figure 6:**
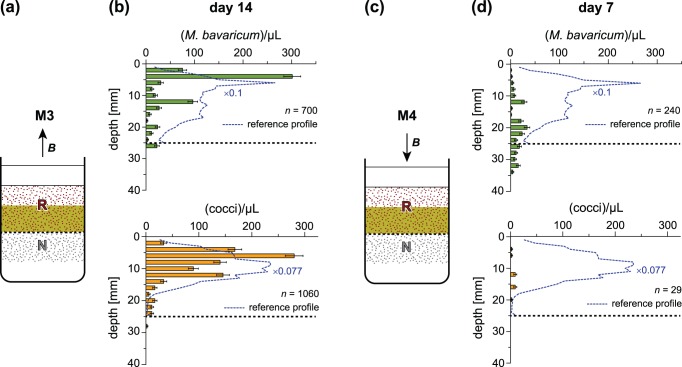
Results of microcosm experiments M3 and M4. R-cells were initially located in their preferred living depth range with (A) upward and (C) downward pointing vertical fields. (B) Cell concentration profiles after 14 days in upward field (correct magnetotaxis). (D) Same as (B), after 7 days in downward field (incorrect magnetotaxis). Long-term mean profiles in the Earth magnetic field obtained from a different microcosm (Figure 5) are shown for comparison (dashed curves), after rescaling cell counts to match concentrations observed in this experiment during correct magnetotaxis. Total MTB populations *n*, obtained by integrating cell counts in each depth, are expressed in 10^6^ cells/m^2^.

The sediment interface of this microcosm is located at 25 mm depth, R cells in the upper layer are within their preferred living range, and ***B*** points upwards (Figure 6a). This experiment reproduces the normal conditions existing in the Southern hemisphere, except for the fact that R-cells have been created by switching the magnetic moment of N-cells. Vertical migration across the sediment boundary is not expected. At the same time, magnetotaxis is incorrect for N-cells, whose number is expected to decline with time. The vertical distribution of R-cells 14 days after microcosm preparation did not change significantly (Figure 6b), and only few R-cells have been found immediately below the interface between the two sediment layers. On the other hand, N-cells of both MBV and MCC were hardly detectable after 14 days (not shown). Experiment results are compatible with the polar M-A model. Furthermore, R-cells created by magnetic moment switching appear to behave exactly like SS cells naturally occurring in the Southern hemisphere, thereby excluding any chemical sensing polarity.

### 4. Microcosm M4: R-cells at preferred depth, incorrect magnetotaxis

This is the same case as M3, up to a downward pointing magnetic field in which R-cells possess the wrong magnetotaxis polarity. In fact, microcosm M3 has been used for this experiment, after reversing the direction of the magnetic field. The initial condition of M4 is therefore given by Figure 6b. After exposure to a downward pointing field for 7 days, R-cell counts decreased markedly for MBV, while MCC became practically extinct (Figure 6d). The MBV population decrease is particularly evident in the topmost 10 mm, where HO cells would migrate upwards because of the incorrect magnetotaxis configuration. On the other hand, R-cells located near the sediment interface, which can be assumed to be in a LO state, are expected to migrate downwards, as seen by their appearance in the bottom sediment layer. Overall, these results can be explained by polar M-A. MBV appears to be more resilient than MCC to incorrectly polarized magnetotaxis.

### 5. Microcosm M1: R-cells in LO state, correct magnetotaxis

**Figure 7:**
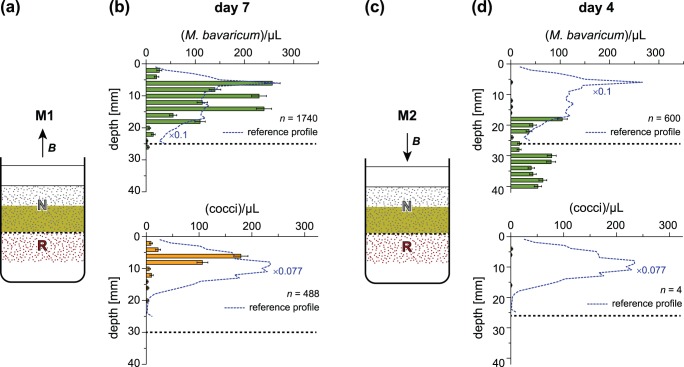
Results of microcosm experiments M1 and M2. R-cells were initially located below their preferred living depth range with (A) upward and (C) downward pointing vertical fields. (B) Cell concentration profiles after 7 days in upward field (correct magnetotaxis). (D) Same as (B), after 4 days in downward field (incorrect magnetotaxis). Long-term mean profiles in the Earth magnetic field (Figure 5) are shown for comparison (dashed curves), after rescaling cell counts as in Figure 6. Total MTB populations *n*, obtained by integrating cell counts in each depth, are expressed in 10^6^ cells/m^2^.

In this experiment, R-cells are initially confined in the bottom sediment layer, at >25 mm depths, and exposed to an upward-pointing field (Figure 7a). The initial depth range is below the preferred one, so that R-cells are expected to migrate upwards, assisted by correctly polarized magnetotaxis. This experiment aimed at testing the swimming direction of polar magnetotaxis under LO conditions, which was not unequivocally determined by anoxic hanging drop assays (section 1). The depth distribution of R-cells 7 days after microcosm preparation confirms that MTB were able to migrate back to their preferred living depth without losses (Figure 7b). Few R-cells appeared in the top layer already after 2 days and almost no R-cells remained in the bottom layer after 7 days. This result confirms the fact that polar MTB switch their swimming direction when exposed to LO conditions. The ∼25 mm migration distance deduced from depth distributions is much larger than the ∼1 mm minimum distance over which magnetotaxis is expected to be the dominant displacement mechanism in sediment [17]. Therefore, a mean vertical migration >3.6 mm/day can be deduced from the fact that ∼25 mm were covered in <7 days. For comparison, migration velocities of the order of 10 mm/day have been predicted with a biased random walk model and 1% magnetic alignment [17].

### 6. Microcosm M2: R-cells in LO state, incorrect magnetotaxis

Microcosm M2 was prepared from M1 after adding a new 25 mm-thick sediment layer containing only N-cells (Figure 7c). This experiment reproduces the same situation of M1, but ***B*** now points downwards, providing an incorrect magnetotactic polarity to R-cells. With this configuration, R-cells in LO state are expected to migrate further down and decrease in number, while no R-cells are expected to appear in the top layer.

MTB profiles taken after 4 days from microcosm preparation (Figure 7d) are consistent with M-A model predictions with respect to the total population, whose decrease is more evident for MCC. On the other hand, some R-cells of MBV migrated upwards into the top layer, against the direction imposed by magnetotaxis on cells in LO state. The ∼12 mm migration distance is sufficiently long for being dominated by magnetotaxis, and corresponds to a velocity of ∼3 mm/day, which is similar to the estimate obtained with microcosm M1. Upward migration of MBV R-cells cannot be explained by current polar M-A models. If R-cells were in a LO state, as expected from the sediment interface depth, a spontaneous conversion of R-cells into N-cells must have occurred by switching the flagellar rotation senses corresponding to LO and HO states, respectively. On the other hand, the existence of HO cells ∼25 mm below the sediment-water interface would be difficult to explain, because the starting point of this experiment was a stable MTB population in its preferred living depth, which soon evolved to LO conditions after addition of the top sediment layer and consequent upward migration of the OAI.

### 7. Microcosm M7: R-cells in HO, correct magnetotaxis

**Figure 8:**
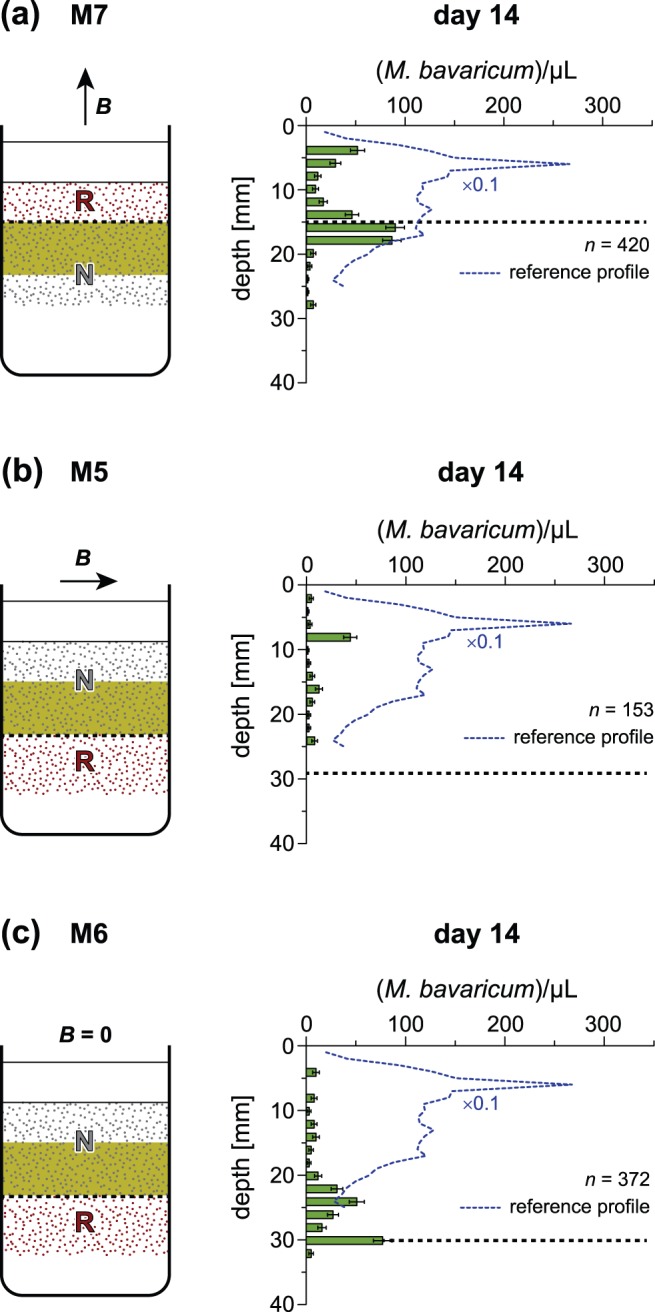
Results of microcosm experiments M7, M5 and M6. (A) Microcosm M7: R-cells of MBV were initially located above their preferred living depth, (<15 mm), in an upward pointing field (correct magnetotaxis of HO cells). (B) Microcosm M5: MBV R-cells were initially located below the preferred living depth (>30 mm), in a horizontal field (indifferent magnetotaxis of LO cells) (C) Microcosm M6: MBV R-cells were initially located below the preferred living depth (>25 mm), in zero field (LO cells with no magnetotaxis). The cell concentration profiles are shown after 14 days from experiment begin, together with long-term mean profiles in the Earth magnetic field (dashed curves), after rescaling cell counts as in Figure 6. Total MTB populations *n*, obtained by integrating cell counts in each depth, are expressed in 10^6^ cells/m^2^.

This experiment is in principle identical to the one performed with microcosm M3, with the only difference that the R-cell-containing top layer has been made as thin as possible (15 mm, Figure 8a), in order to monitor the migration of MBV cells in their HO state, assisted by a correct magnetotaxis polarity. After 14 days, high concentrations of R-cells were detected in vertical profiles down to 20 mm, and few cells have been found down to 27 mm (profile in Figure 8a). This experiment demonstrates the vertical migration of HO cells, as predicted by the M-A model. The concentration of N-cells, on the other hand, decreased significantly (data not shown), due to incorrectly polarized magnetotaxis.

### 8. Microcosm M5: R-cells in LO, horizontal field

This experiment aims at testing M-A in horizontal fields, as it would naturally occur at the geo­magnetic Equator. Microcosm preparation was identical to M1, except that R-cells in LO state inside the bottom layer are exposed to a horizontal field (Figure 8b). In this case, magnetotaxis does not provide any preferred direction for vertical migration (indifferent magnetotaxis). The M-A model, combined with low magnetic alignments, predicts that any vertical migration of LO cells relies only on chemotaxis. N- and R-cells of MCC, though being abundant at the beginning of the experiments, disappeared completely after 14 days (data not shown). R-cells of MBV, on the other hand, migrated into the top layer (profile in Figure 8b) at cost of a significant population decrease. For comparison, vertical migration of LO cells assisted by a correctly oriented vertical field (microcosm M1) occurred without significant cell count decreases.

### 9. Microcosm M6: R-cells in LO, no magnetotaxis

This experiment explores the vertical migration capability of LO cells when magnetotaxis is elimi­nated by cancellation of the local magnetic field. In this case, MTB cells rely only on chemotaxis. R- and N-cells of MCC disappeared after 14 days, while R-cells of MBV migrated into the top layer without a significant population decrease (Figure 8c). The disappearance of MCC apparently contradicts the long-term resilience experiment (section 2) where the same type of bacteria was practically unaffected during >6 months in zero field. However, the resilience experiment has been performed with undisturbed sediment in which cells where not displaced from their preferred depth, as it is the case here.

## Discussion

Experiments discussed in the results chapter are conveniently grouped according to (1) the initial MTB distribution with respect to the preferred living depth (i.e. at, above, and below) and (2) the magnetotactic configuration (i.e. ‘correct’ and ‘incorrect’ magnetotaxis in vertical fields, ‘indifferent’ magnetotaxis in horizontal fields, and no magnetotaxis in zero field). Results (Table 2) are expressed in terms of detectable vertical displacements (i.e. R-cells appearing in a sediment layer where they were not originally present) and evolution of total populations over time. Because cell counts are subjected to large uncertainties due to microcosm heterogeneity (i.e. lateral profile variations) and temporal fluctuations, population changes are considered significant only if they exceed a given factor that depends on the type of experiment. In case of resilience experiments (Table 1) the large number of measured profiles enabled the application of a Kolmogorov-Smirnov test for two variables with unknown probability distributions (i.e. total MBV and MCC populations subjected to normal and special field configurations, respectively). In all vertical migration experiments (microcosms M1–M7), population changes are considered significant if they exceed a factor of 2. This factor has been chosen because it corresponds to the maximum cell count standard deviation of 88 individual sediment profiles taken under identical, stationary conditions (Figure 1c). Furthermore, a MTB population is considered nearly extinct if only 1–2 cells were counted in each depth interval.

**Table 2:** Summary of microcosm experiment results.

Condition	Correct MT	Incorrect MT	Indifferent MT	No MT	Alternating MT
**Above PD**	**M7**	–	–	–	–
Migration	↓ MBV				
Population	↔ MBV				
**At PD**	**M3**	**M4**	–	**R0**	**RA**
Migration	↔ all	↓ MBV (partly)		↔ all	↔ all
Population	↔ all	↓ all		↓ MBV, ↔ MCC	↓ MBV, 0 MCC
**Below PD**	**M1**	**M2**	**M5**	**M6**	–
Migration	↑ all	↑ MBV	↑ MBV	↑ MBV	
Population	↔ all	↓ MBV, 0 MCC	↓ MBV, 0 MCC	↔ MBV, 0 MCC	

Abbreviations: PD = preferred depth, ↑ = upward migration, ↓ = downward migration or significant population decline, ↔ = no significant population decline or no vertical migration, 0 = disappearance (down to 0–1 counts), R0 = resilience in zero field, RA = resilience in alternating field.

Vertical displacements give information about how magnetotaxis is combined with chemical sensing, while a significant population decrease can be interpreted as the consequence of unfavourable envi­ronmental conditions that cannot be efficiently escaped. Experiment results appear contradictory with respect to the existence of a magnetotactic advantage in sediment: for example, resilience experiments in zero fields clearly demonstrate this advantage for MBV, but not for MCC, while a correct field polarity is essential for enabling displaced MTB cells of both types to reach their preferred depth. Furthermore, some experiments (e.g. M2) revealed the ability of MBV to migrate against the direction imposed by magnetotaxis. Overall, MBV and MCC appear to use magnetotaxis in a different manner. In order to gain some systematic understanding of our experiments, we summarized results according to the capability of MBV and MCC to cope with specific situations summarized in Table 3, which are discussed in the following.

**Table 3:** Summary of observed MTB behaviours under different magnetic field configurations.

MTB ability to:	Key experiments	*M. bavaricum*	Cocci
Use correct magnetotaxis when undisturbed	R0	Yes	No
Use correct magnetotaxis when displaced	M1 compared to M2, M5, and M6	Yes	Yes
Use chemotaxis when displaced in zero field	M6	Yes	No
Override incorrect magnetotaxis when displaced	M2 and RA	Yes	No

### 1. Can MTB rely only on chemotaxis?

Zero-field experiments with microcosm M6 prove the capability of MBV to rely only on chemotaxis for reaching the optimal living depth in sediment. Similar results have also been obtained in a horizontal field (microcosm M5), where the absence of a vertical field component is equivalent to zero-field conditions as far as vertical migration is concerned. The capability of chemical reception to modify the MTB swimming behaviour (specifically by switching flagellar rotation) has been clearly demonstrated by M-A experiments with cultured cells [5]. However, polar M-A is not expected to support macroscopic displacements in zero field, because HO/LO cells would swim at random directions without run-and-tumble, run-reverse, or run-reverse-flick behaviours typical of non-magnetic bacteria performing chemotaxis [24], [25]. The common feature of these chemotactic swimming behaviours is that nearly straight paths are interrupted by sharp directional changes, whose frequency is determined by the chemical environment (i.e. more frequent changes in presence of repellents). Directional changes are essential for introducing a chemically controlled, systematic bias to an otherwise fully random displacement.

Systematic observations of >1000 swimming trajectories of MBV and MCC with the hanging drop assay in precisely controlled zero fields did not reveal a single case where the continuity of (random) swimming paths was interrupted by tumbling or reverse-flick events [17]. On the other hand, MBV sometimes displays a tactile response when colliding with sediment particles in hanging drop assays prepared with diluted sediment (video S2). Tactile responses consist in brief stops once a sediment particle is hit, as well as swimming direction reversals for variable amounts of time ranging from 1 s to the longest observation time (∼10 s). Such tactile responses do not occur systematically and might therefore depend on some internal state of the cell. On the other hand, tactile responses could never be observed with MCC under identical conditions. Hanging drop observations have been performed in a magnetic field, which is required for ‘extracting’ MTB from opaque sediment, and then make cells swim back into sediment by reversing the field direction. Under such conditions, ‘backward’ swimming of MBV is characterized by a pronounced wiggling and ∼30% speed reduction. The asymmetry between forward and backward swimming comes from the fact that, during forward swimming, flagella are arranged into a single, coherent bundle forming a helix that spirals around the body, minimizing viscous drag [26]. Reversing the sense of flagellar rotation make individual flagella sprout outwards, providing incoherent contributions to a less efficient propulsion. If an aligning field is absent, reversed flagellar rotation produces sudden cell reorientation by random angles, similar to those observed during chemotaxis (video S3). The fact that only MBV is able to rely exclusively on chemotaxis for macroscopic vertical displacements might be related to its ability to briefly reverse flagellar rotation even when being in a definite HO/LO state. This mechanism can be understood as a dominantly polar M-A with an axial component triggered by specific stimuli.

### 2. Is magnetotaxis used for overcoming vertical displacement from the optimal living depth?

The answer to this question seems obvious when considering that this should be the primary function of magnetotaxis. On the other hand, the poor (<1%) magnetic alignment of MTB in sediment [17] might nullify any magnetotactic advantage in this environment, leaving magnetotaxis as a guide for guiding the bacteria, when dislodged, back to the sediment, as postulated in [4]. We have investigated sedimentary magnetotaxis by monitoring the vertical migration of displaced R-cells under the following three field configurations: (1) ‘correct’ (*B_z_*>0), (2) ‘incorrect’ (*B_z_*<0), and (3) ‘none’ (**B** = 0) or ‘indifferent’ (*B_z_* = 0). Vertical migration occurs with no cell count decrease in case of correct confi­gurations for both MBV and MCC. On the other hand, MCC are intolerant to incorrect field orientations, while MBV appears to be able to migrate towards the preferred living depth even against the direction dictated by magnetotaxis, albeit at cost of a significant population decrease. This is only possible if an axial magnetotaxis mechanism somehow overrides polar M-A when necessary. The same capability is seen with resilience experiments, where the daily field polarity change was fatal to MCC but not to MBV. Finally, vertical migration of MBV in correct field configurations (25 mm in ≤7 days) appears to be faster than vertical migration in zero field (20 mm in ≤14 days). Overall, our experiments demonstrate the existence of a magnetotactic advantage supporting vertical migration inside sediment, despite poor magnetic alignment. This advantage seems an essential condition for survival of displaced MCC cells, while MBV is able to migrate without a magnetic field and even against the direction imposed by polar M-A.

### 3. Is magnetotaxis used within the optimal living range?

Zero-field resilience experiments with undisturbed sediment show that MBV and MCC populations can survive without magnetotaxis if they were originally located within their preferred living range. Total MCC cell counts did not change significantly in zero field, while MBV decreased initially by 50% and remained stable at this level for 6 months. This result is unexpected when considering that displaced MBV cells have a much higher capability of migrating back to their preferred living depth in comparison to MCC. The apparent contradiction between experimental results with displaced and undisplaced MTB populations can be explained if magnetotaxis is somehow continuously used by MBV, even within the preferred living range, while it plays a minor role in case of undisturbed MCC populations. For example, MBV can take advantage from magnetotaxis if it needs to shuttle between oxic and anoxic sediment levels for satisfying different metabolic requirements, as postulated by [27]. This mechanism, called ‘redoxtaxis’, has been introduced to explain some morphological peculiarities of MBV cells, such as large size and the presence of sulphur inclusions that would be filled when plunging deep in sediment and oxidized after moving into more superficial layers. Empty and filled inclusions can be clearly seen as lighter and darker areas in TEM micrographs, sometimes coexisting within the same cell (e.g. Figure 1d).

The redoxtaxis hypothesis is indirectly supported by our experiments in several ways. First, the bi­modal depth distribution of MBV is compatible with cells spending most time at depths where sulphur oxidation can take place (shallower peak at ∼5 mm), and depths where sulphur is accumulated (deeper peak at ∼16 mm). Second, vertical shuttling between these depths occur over macroscopic ranges (∼11 mm) largely exceeding the ∼1 mm minimum distance required for magnetotaxis to become effective in case of <1% alignments with the Earth magnetic field. For comparison, microscopic displacements required by MTB living at a single preferred depth for contrasting slow sediment mixing occur over distances where magnetotaxis is less effective, explaining the insensitivity of undisturbed MCC populations to the lack of a magnetic field. On the other hand, incorrect field polarities trigger a runaway mechanism by which displaced cells tend to move in the wrong direction.

## Conclusions

The magnetotactic advantage of two wild MTB types living in sediment – undefined round cocci and *M. bavaricum* – has been investigated with experiments aimed at testing the capability of (1) undisturbed populations to survive zero-field and field reversal conditions, and (2) displaced populations to migrate back to their preferred living depths under different field configurations. These experiments confirmed that a magnetotactic advantage exists for both MTB types if they are displaced from preferred living depths, for example by bioturbation, even if their mean alignment with the Earth magnetic field is <1%. On the other hand, many responses of the two MTB types to our experimental conditions were clearly distinct and partially unexpected on the basis of current polar M-A models. For example, MBV can migrate over macroscopic distances in zero field, thus relying exclusively on chemotaxis, while this is impossible for MCC. Displaced MBV cells can even override wrongly oriented fields, although at cost of a population decrease.

The ability of MBV to rely only on chemotaxis for macroscopic displacements can be explained by facultative tactile responses of cells colliding with sediment particles. Such responses, as observed in hanging drop assays in a magnetic field, include swimming reversals lasting for variable amounts of time, which, in zero field, might produce the ‘tumbles’ required by magnetotaxis. It is important to realize that these reversals have been observed on cells with a clearly defined HO state, and should therefore not be confused with HO-LO transitions in polar M-A. This type of reversals is also required to explain the capability of displaced MBV populations to migrate towards their preferred living depth against the direction dictated by polar M-A. We tentatively explain these observations with the possibility for MBV to switch between polar and axial M-A. Cells with a defined HO/LO state would maintain a dominant magnetotaxis polarity for a certain period of time. If HO/LO conditions persist over this time, magnetotaxis polarity would be reversed, becoming de facto part of an axial magnetotactic mechanism. The delayed onset of this mechanism would enable MBV to profit from specific advantages of polar and axial magnetotaxis, i.e. the insensitivity of axial magnetotaxis to the direction of chemical gradients with respect to the magnetic field, and the insensitivity of polar magnetotaxis to small-scale gradient fluctuations in which cells using ‘fast’ axial M-A would easily become trapped. MCC do not possess this ability, and appear to rely only on polar magnetotaxis.

Another unexpected difference between MBV and MCC is observed when stable populations living in undisturbed sediment are kept in a zero-field environment for long periods of time. In this case, MBV populations decrease by ∼50%, while no changes are observed for MCC. This difference can only be explained by different mobility requirements during stationary conditions. As previously proposed [27], MBV would continuously shuttle between sediment layers with different chemical properties for satisfying metabolic requirements (redoxtaxis), while cocci might seek a single preferred living depth, becoming less motile when this condition is satisfied. In this case, MCC would use magnetotaxis only for overcoming macroscopic displacements produced by sediment bioturbation.

Results of this study suggests that magnetotaxis might be more complicated than originally implied by the M-A model, and that mechanical interactions with sediment particles could play an important role. Furthermore, different magnetotaxis ‘styles’ might represent a response to specific biological require­ments. In this context, direct MTB observations in aqueous media provide only a very limited insight into complex interactions between magnetotaxis, chemical sensing, and sediment heterogeneities.

## Supporting Information

Table S1 **Data.** This table contains all experimental data underlying Figures 1–8.
(XLS)

Text S1 **Detailed description of experimental settings.** Section 1: hanging drop assays. Section 2: resilience experiments. Section 3: microcosm preparation for vertical migration experiments.
(PDF)

Video S1 **Hanging drop observations of** ***M. bavaricum*** **and cocci.** Hanging drop assay showing *M. bavaricum* and unspecified magnetotactic cocci accumulating along the right edge of a water drop. The local magnetic field generated by Helmholtz coils is parallel to the observation plane and points to the right, as indicated by the white line inscribed in a circle. Therefore, MTB cells accumulating on the right edge are north-seeking (NS). *M. bavaricum* is easily distinguishable from cocci because of its elongated shape and larger size. During the first two parts of the video, few *M. bavaricum* cells, already immobilized in the partially dried drop edge, are surrounded by highly motile cocci performing backward loops not observed in case of freely swimming cells. The third part of the video shows another region of the drop edge with more, still motile *M. bavaricum* cells. The last part of the video show *M. bavaricum* and cocci swimming away from and back to the drop edge after reversing the field direction twice.
(MOV)

Video S2 **In-field tactile response of** ***M. bavaricum*** **when colliding with sediment particles.** Hanging drop assay prepared with higher sediment concentration for observing interactions of *M. bavaricum* cells with particles. The magnetic field points to the right. Tactile responses can be seen on freely swimming cells coming from the left. Some cells briefly reverse their swimming direction upon colliding with sediment particles (e.g. large cell near the center at 00′03″, and large cell on the bottom after at 00′37″). Other cells engage a long-lasting series of swimming direction reversals, oscillating around a mean position in proximity of sediment particles. These behaviors are never observed in absence of sediment particles. The field direction is reversed at 00′46″. Most cells that have accumulated on the right drop edge cross the region occupied by sediment particles without changing their swimming behavior, as a demonstration that tactile responses are facultative. A large cells swimming to the left near the video bottom stops its motion abruptly after touching a sediment particle (00′47″).
(WMV)

Video S3 **In-field and zero-field swimming behavior of** ***M. bavaricum*** **in a sediment-rich, aqueous environment.** Hanging drop assay prepared with higher sediment concentration for observing the swimming behavior of *M. bavaricum* cells. The magnetic field points to the left until 00′09″, and zero-field conditions are established afterwards, with a brief interruption during 00∶49–56″. Frequent in-field swimming direction reversals are related to the presence of sediment and are not observed in pure water. After establishing zero-field conditions, cells swim at random directions along nearly straight path interrupted by sudden direction changes according to the modes described in [24]. Such direction changes have been never observed in in hanging drop assays with pure water [16]. Some events are highlighted in the following. 00′20–25″ center-top: cell swims upwards, enter a sediment cluster and exits after a ∼180° turn deduced from the fact that the cell is still moving forward (absence of cell wiggling characteristic for backward swimming). 00′26–27″ center-right: cell reverses flagellar rotation and swims backward (wiggling) after coming close to an isolated sediment particle. 00′27–30″ center-right: cell ‘collides’ with a sediment particle, rotates ∼45°CW, swims backwards for a short time, and finally rotates ∼45°CCW after reversing the swimming direction a second time. This event corresponds to a ‘run-reverse-flick’ pattern [24]. 00′28–33″ bottom-right: cell collides with a sediment particles and reverses swimming direction (∼00′30″). While swimming backwards, it collides with another swimming cell (∼00′31″). During this collision, the swimming direction is reversed a second time after short tumbling corresponding to a ‘run-and-tumble’ pattern [24]. 00′33–37″ top-right: cell starts to tumble chaotically just after passing by a couple of sediment particles. 00′42–46″ center to top-left: sequence of two run-reverse-flick patterns [24] triggered in proximity to sediment particles.
(MP4)
