# Supplementary material for: Magneto-Chemotaxis in Sediment: First Insights
Source: PLoS One. 2014 Jul 17;9(7):e102810. doi: 10.1371/journal.pone.0102810 (PMC4102565; doi:10.1371/journal.pone.0102810)
Supplement: Text S1 — Detailed description of experimental settings. Section 1: hanging drop assays. Section 2: resilience experiments. Section 3: microcosm preparation for vertical migration experiments. (PDF) [file pone.0102810.s002.pdf]

# Magneto-chemotaxis in sediment: First insights

Xuegang Mao [1,3], Ramon Egli [2], Nikolai Petersen [3], Marianne Hanzlik [4], and Xiuming Liu [1,5]

1. College of Geographical Sciences, Fujian Normal University, Fuzhou, China.
2. Corresponding author. Central institute for Meteorology and Geodynamics, Vienna, Austria. E-mail: ramon.egli@zamg.ac.at.
3. Department of Earth and Environmental Sciences, Ludwig-Maximilians University, Munich, Germany.
4. Chemistry Department, Munich Technical University, Garching, Germany.
5. Department of Environment and Geography, Macquarie University, Sydney, Australia.

# Supporting text S1

# 1. Experimental settings for observing MTB in air and anoxic condition

## 1.1 Hanging drop assays in air

The apparatus for observing MTB (so-called bacteriodrome) consists of an optical microscope whose magnetic parts have been removed or replaced as far as possible (Figure 1). The microscope is surrounded by two pairs of electronically driven Helmholtz coils for generating a controlled homogeneous field over the observation plane, and is equipped with a CCD camera for real-time recording of images and magnetic field direction/intensity.

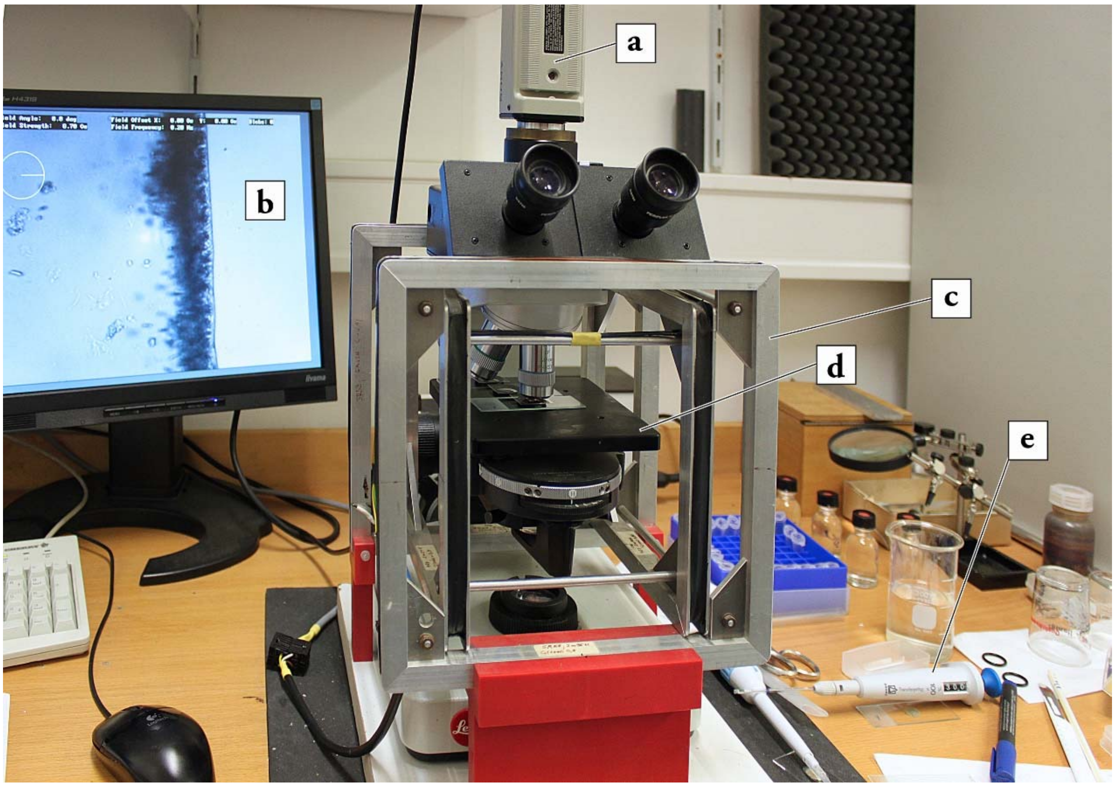


**Figure 1.** Apparatus for the observation of magnetotactic bacteria (bacteriodrome). (**a**) Camera attached to the optical microscope. (**b**) Live image from camera, showing *M. bavaricum* cells accumulating at the edge of the water drop in the hanging drop assay. (**c**) Helmholtz coils for providing controlled fields in horizontal direction. (**d**) Object stage. (**e**) Micropipette.

Hanging drop assays are prepared as follows. A drop of sediment slurry is placed on a cover slide and diluted with distilled water, so that MTB-containing sediment slurry is located at the center of a clear water drop (Figure 2). The cover slide is placed upside-down onto a plastic O-ring resting on a microscope slide. The water drop is thus in enclosed in a space sealed by the O-ring, which prevents evaporation, enabling >30 min observation time under the microscope. MTB leave the sediment slurry and swim towards the water drop edge according to their swimming polarity (i.e. N- or S-seeking) in the magnetic field generated by the Helmholtz coils of the bacteriodrome. The hanging drop is placed in the bacteriodrome (Figure 1) in a horizontal magnetic field, allowing the bacteria move to the drop edge. Because new MTB are no longer leaving the sediment after 20 min., *M. bavaricum* and cocci are counted after 20 min. exposure in the magnetic field.

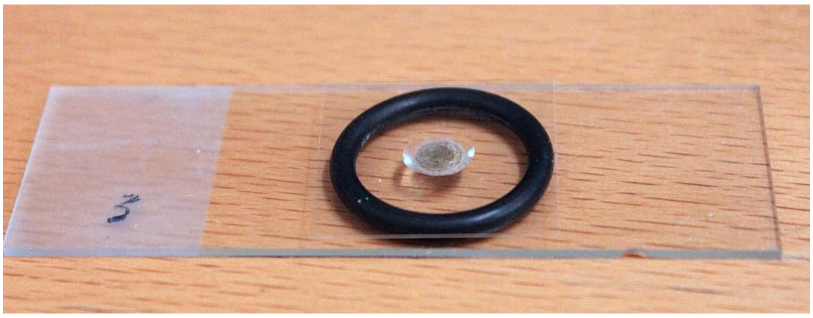

Figure 2. Hanging drop ready for observation. The cover slide carrying a hanging drop is placed onto an O-ring (black) resting on a microscope slide.

### 1.2 Hanging drop assays under anoxic conditions

Anoxic hanging drop assays have been performed with same procedures described in section 2.1, with all materials (microcosm, bacteriodrome, microscope slides) placed inside a sealed glove box (Figure 3). The glove box was constantly flushed with ~1 L/min nitrogen gas bubbled trough a saturated $FeCl_2$ solution for residual oxygen removal. A slight overpressure was maintained in order to prevent inward air diffusion. All materials and consumables required for the hanging drop assays were previously placed in the glove box, which remained sealed for the entire experiment duration.

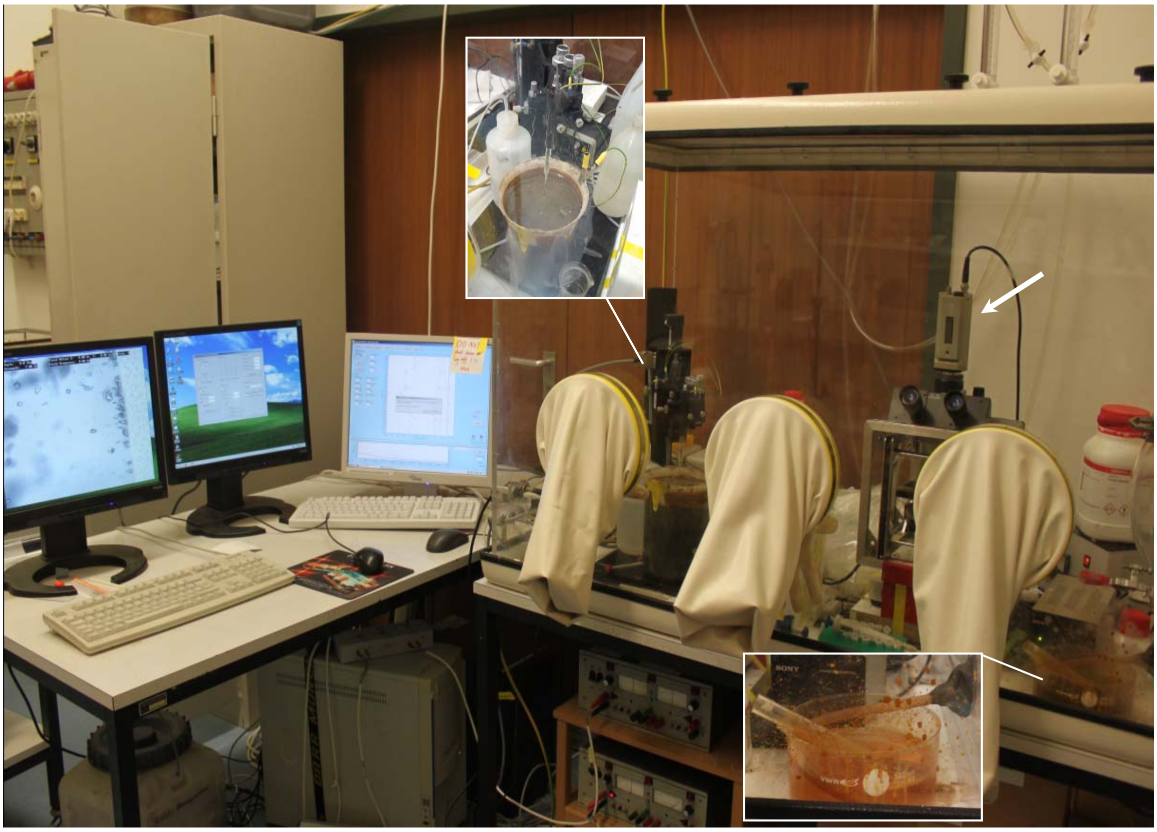

Figure 3. Glove box used for anoxic hanging drop assays (right). Monitors (left) are connected to the bacteriodrome (arrow) and the Unisense motorized oxygen microprofiler through airtight fittings. Inserts show the microcosm placed under the oxygen microprofiler inside the glove box (top), and the $FeCl_2$ solution through which nitrogen was bubbled (bottom). The solution was fully oxidized one week after at the end of the experiment, when the picture was taken.

## 2. Resilience experiments

The experimental setup for the resilience experiments and maps of the residual field during zero-field periods are shown in Figure 4 and Figure 5, respectively.

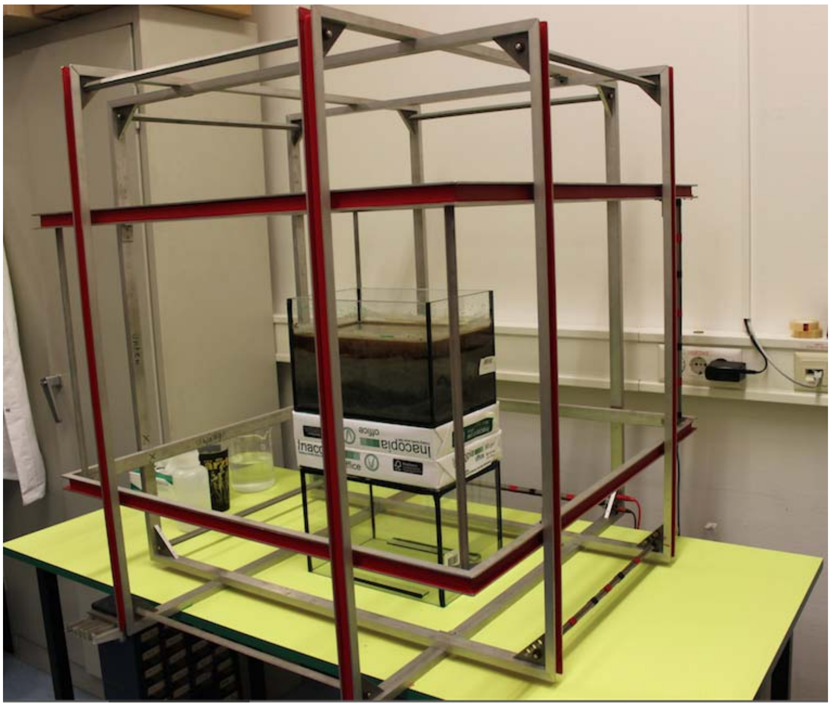

Figure 4. Experimental setup for long-term resilience experiments. A sediment-filled 30×20×20 cm glass aquarium was placed at the center of three ~1×1 m square Helmholtz coil pairs.

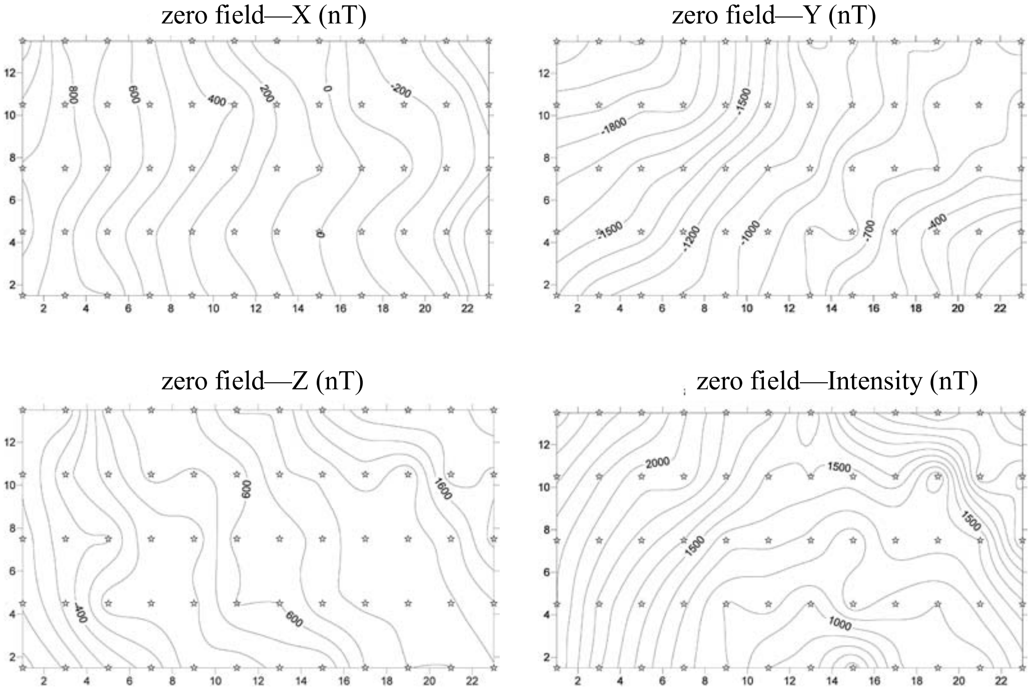


Figure 5. Map of measured residual fields over the space occupied by the aquarium at the beginning of the zero-field experiment. Numbers on horizontal and vertical axis are coordinates in cm. Stars in the maps represent measurement points.

An upper limit for a possible magnetotactic bias during zero-field experiments is estimated by assuming that a hypothetical systematic field residual is <10% of maximum measured values i.e. <200 nT. Assuming 1% to be the upper limit for the mean alignment of MTB with Earth-like magnetic fields (i.e. $B \approx 50$ μT) in sediment [16], a simple proportion gives a ~250 times worse alignment in 200 nT. Accordingly, magnetotaxis efficiency during zero-field resilience experiments is <0.5% of the efficiency expected under natural conditions and can be considered negligible.

## 3. Preparation of sediment microcosms for vertical migration experiments

Preparation of sediment microcosms consisting of two sediment layers with sharp interface is illustrated in Figure 6 for the case where R-cells are located in the bottom layer. The starting point is a microcosm with stable chemical stratification (Figure 6a). A pair of coils for the generation of strong field pulses (~0.1 T for ~50 μs) is immersed in the water column and placed few mm over the sediment-water interface (Fig. 6b). Magnetic pulses are applied repeatedly while moving the coil pair all over the sediment in order to create a homogeneous layer of R-cells. With this procedure, ~50% R-cells are created in the uppermost ~25 mm, where the pulse field strength is sufficiently large for switching favorably oriented magnetic moments. After creating R-cells, sediment containing only N-cells was taken from the same aquarium used for microcosm preparation, and gently deposited over the microcosm sediment (Figure 6c). In order to obtain a sharp interface between the two layers, deposition occurred very slowly by dripping sediment slurry from a sieve through the water column of the microcosm, until the sediment-water interface was raised by ~25 mm. Immediately after deposition of the new layer, the microcosm consists of two sediment layers with R-cells contained exclusively in the bottom layer (Figure 6d). A new chemical stratification is built over 3-4 days, as oxygen is consumed in the bottom layer (Figure 6e). Consequently, R-cells originally located within or above their optimal living depth, are exposed to LO conditions, which stimulate their migration into the top layer originally containing only N-cells.

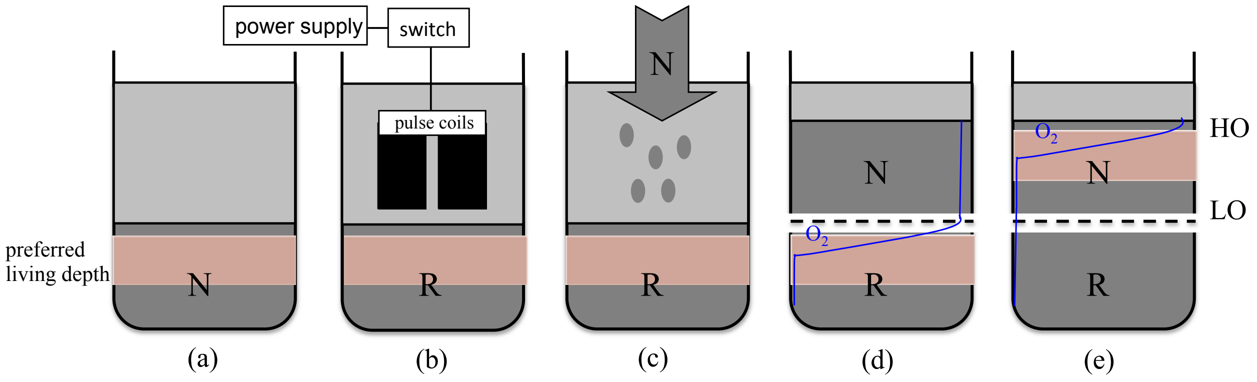


**Figure 6.** Microcosm preparation for vertical migration experiments. (**a**) Starting point at chemical equilibrium. (**b**) Creation of R-cells with magnetic field pulses produced by a coil pair placed immediately above the sediment-water interface. (**c**) Deposition of a new sediment layer containing only N-cells. (**d**) Chemical stratification immediately after microcosm preparation (dashed line: interface between the two sediment layers; blue: typical oxygen profile). (**e**) Chemical stratification after reaching a new equilibrium in 3-4 days. R-cells are now located below their optimal living depth and are in a LO state.
